# Supplementary material for: CloseRead: a tool for assessing assembly errors in immunoglobulin loci applied to vertebrate long-read genome assemblies
Source: Genome Biol. 2025 May 20;26:131. doi: 10.1186/s13059-025-03594-7 (PMC12090573; doi:10.1186/s13059-025-03594-7)
Supplement: Supplementary file 1 — Additional file 1: Figures S1–S8 and Note S1. [file 13059_2025_3594_MOESM1_ESM.pdf]

Supplementary Information

Breaks in Coverage

For breaks with consecutive 'N' reference sequences, which represent unknown bases, these are often linked to complex genomic structures or repetitive elements. This includes cases with one or two reads mapped, where reads may still align if the surrounding sequences provide sufficient context for alignment algorithms to operate despite uncertainties. It also covers breaks with zero reads aligned, referred as gaps in this paper, indicating completely unsequenced or unassembled regions.

In contrast, for non-'N' reference sequences, breaks with fewer than two (non zero) reads mapped suggest regions with sparse sequencing data, potentially highlighting unsupported sequences that might reflect errors in the assembly. Breaks with no reads aligned point to more substantial data gaps, where the reference sequence is known but completely lacks sequencing support, providing stronger evidence of possible assembly inaccuracies.

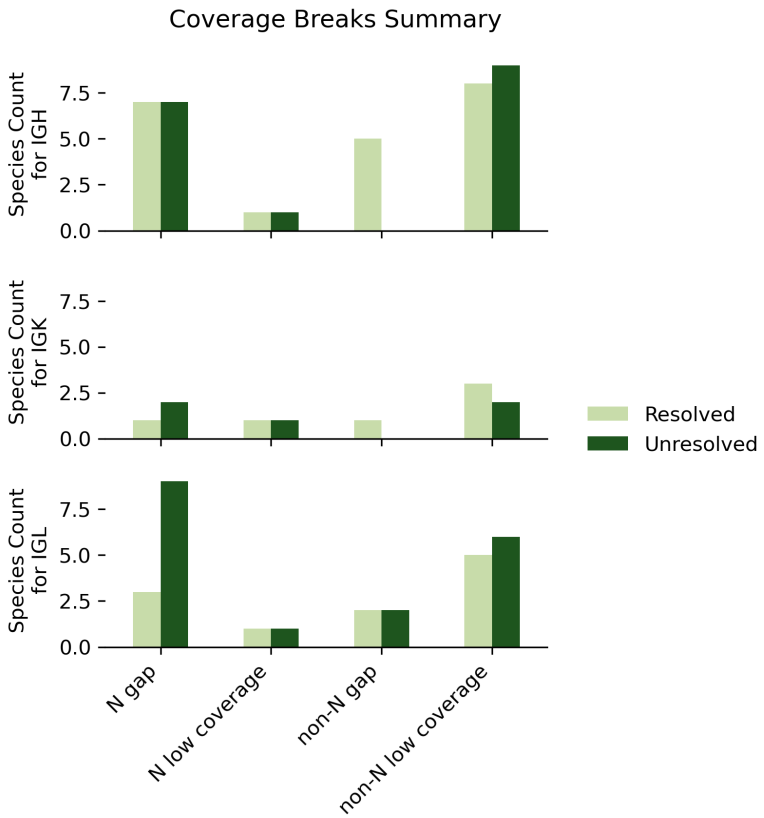

Figure S1. Breakdown of Assembly Break Types Across Species in IGH, IGK and IGL loci. This figure displays the frequency of four distinct types of assembly breaks across various species within the IGH, IGK, and IGL loci. Break types are categorized as follows: N low coverage (fewer than two reads mapped in regions with consecutive 'N' reference sequences); N gap (zero reads mapped in regions with consecutive 'N' reference sequences); non-'N' low coverage (fewer than two reads mapped in regions with non-'N' reference sequence); and

non-N gap (no reads mapped in regions with non-'N' reference sequence). Each bar is color-coded to indicate whether the species' assemblies are haplotype-resolved.

Case Studies Supplementary

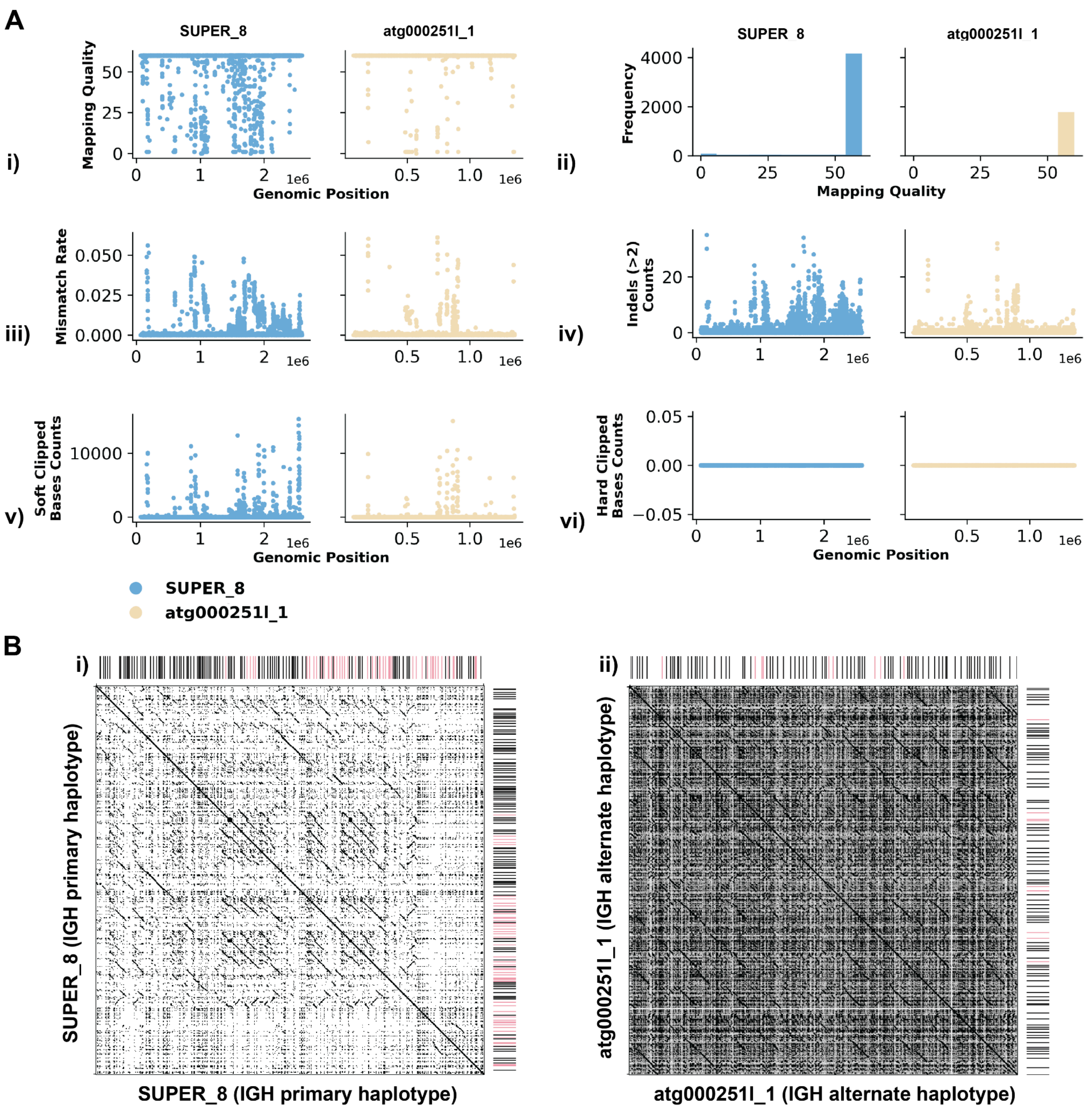

Figure S2. Additional Analysis of IGH Locus Assembly Errors in Greenland wolf (*C. l. orion*). A. Summary statistics of the read alignment situation are depicted, showing mapping quality across IGH loci for both haplotypes, with blue representing the primary assembly and yellow the alternate. i) mapping quality across IGH loci for both haplotypes, with blue representing the primary assembly and yellow the alternate, ii) read mapping quality frequency, iii) mismatch rates of reads, and iv) number of reads with indels of consecutive length of at least

2 bp, v) count of soft clipped bases in each read, vi) count of hard clipped bases in each read, for both haplotypes across IGH loci. B. Dotplots comparing gene locations and alignments are shown for i) primary vs primary and ii) alternate vs alternate haplotypes.

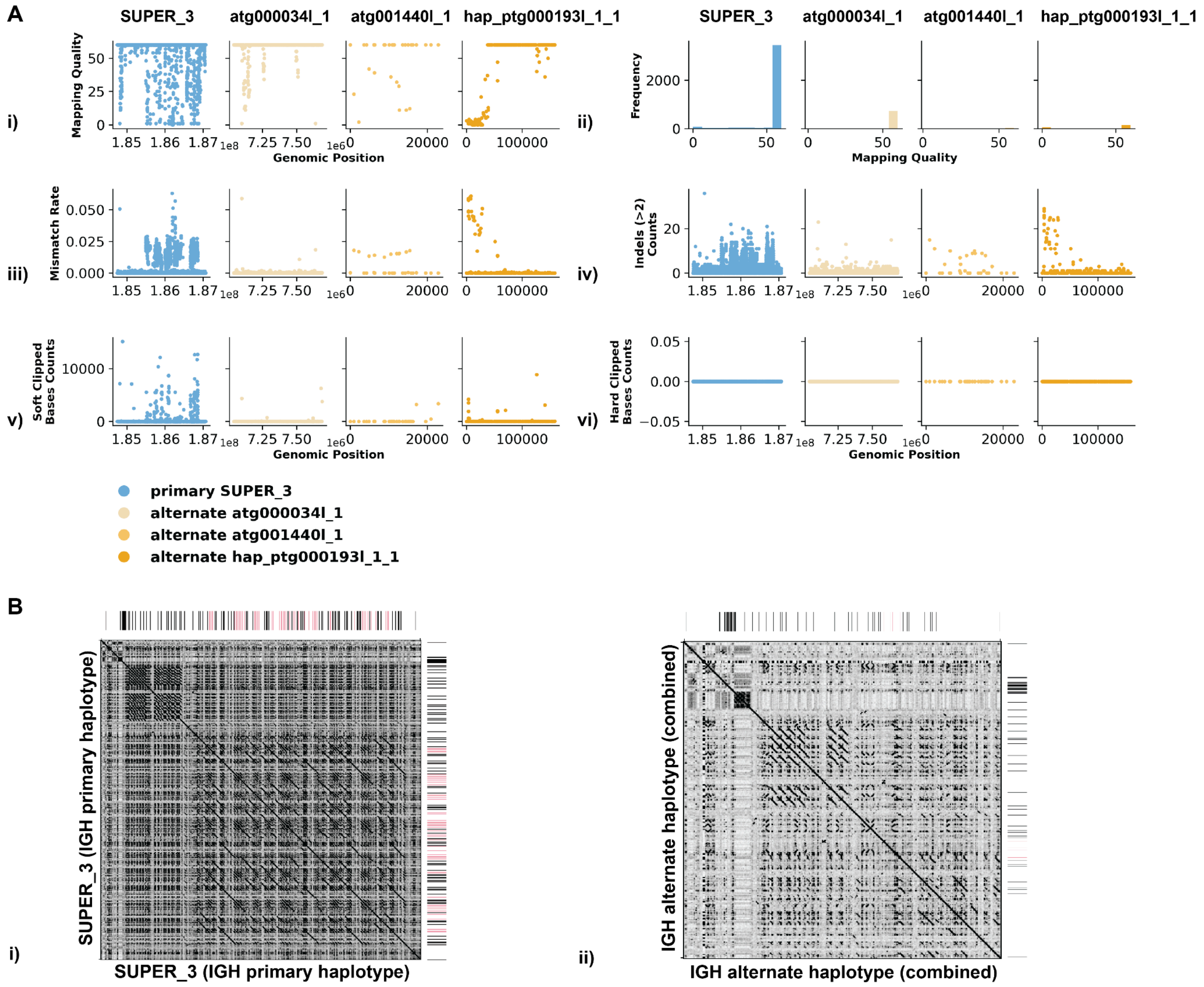

Figure S3. Additional Analysis of IGH Locus Assembly Errors in Philippine Flying Lemur (*C. volans*). A. Summary statistics of the read alignment situation are depicted, showing mapping quality across IGH loci for both haplotypes, with blue representing the primary assembly and yellow the alternate. i) mapping quality across IGH loci for both haplotypes, with blue representing the primary assembly and yellow the alternate, ii) read mapping quality frequency, iii) mismatch rates of reads, and iv) number of reads with indels of consecutive length of at least 2 bp, v) count of soft clipped bases in each read, vi) count of hard clipped bases in each read, for both haplotypes across IGH loci. B. Dotplots comparing gene locations and alignments are shown for i) primary vs primary and ii) alternate vs alternate haplotypes.

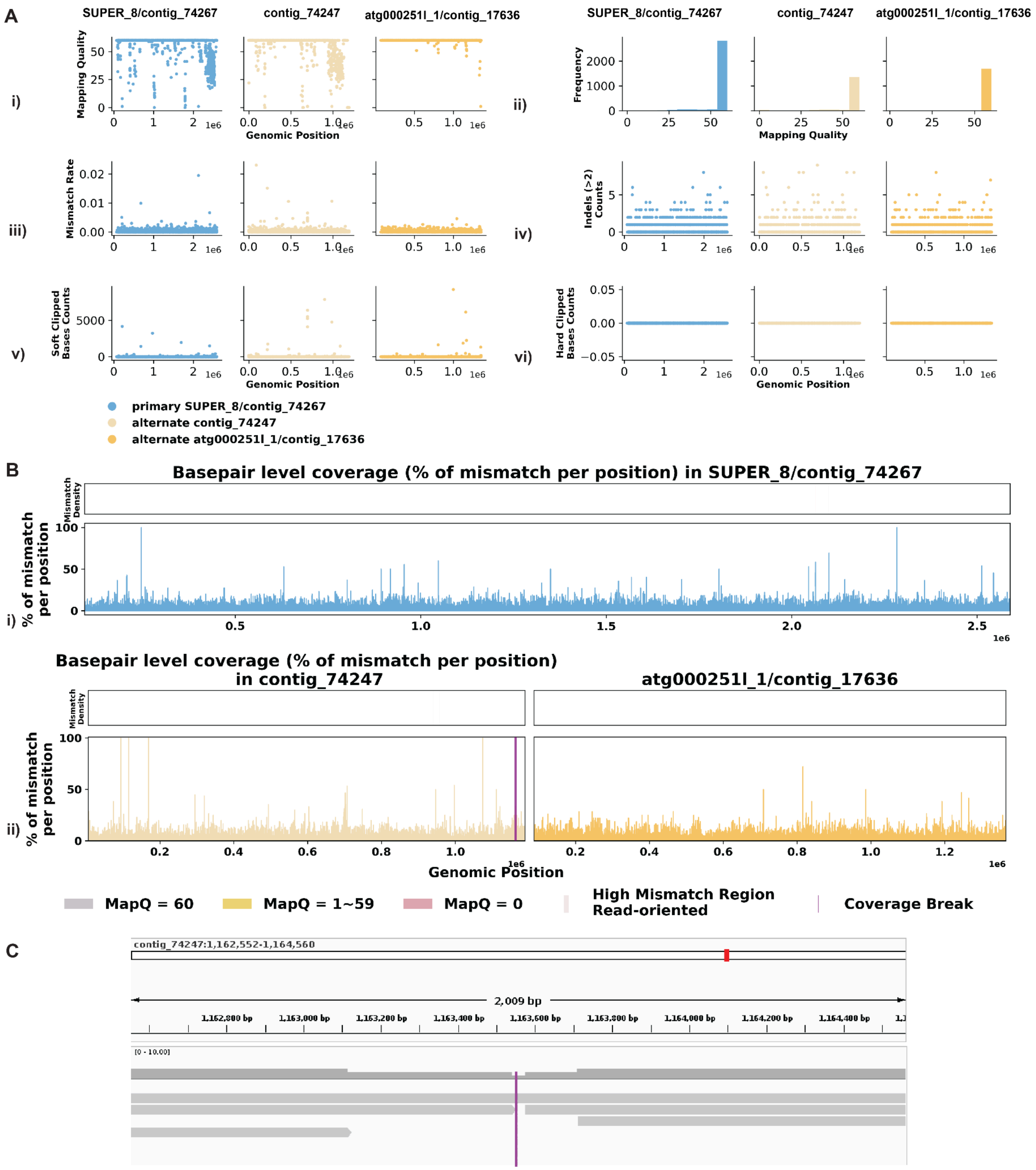

Figure S4. Additional Reassembly Analysis of IGH Locus Assembly Errors in *C. l. orion*. A. Summary statistics of the read alignment situation are depicted, showing mapping quality across IGH loci for both haplotypes, with blue representing the primary assembly and yellow the alternate. i) mapping quality across IGH loci for both haplotypes, with blue representing the primary assembly and yellow the alternate, ii) read mapping quality frequency, iii) mismatch rates of reads, and iv) number of reads with indels of consecutive length of at least 2 bp, v) count of soft clipped bases in each read, vi) count of hard clipped bases in each read, for both haplotypes across IGH loci. B. Reassembly IGH *basepair-oriented* mismatch rate across i) primary haplotype, ii) alternate haplotype; a heatmap above indicating the frequency of high

mismatch rate base pairs, with darker colors denoting more frequent occurrences. Light red highlights positions covered by ≥5 reads with an error rate >1%, and purple bars indicate coverage breaks (coverage ≤2). C. IGV screenshot of the low coverage region in contig “74247”, purple indicate where the break in coverage is.

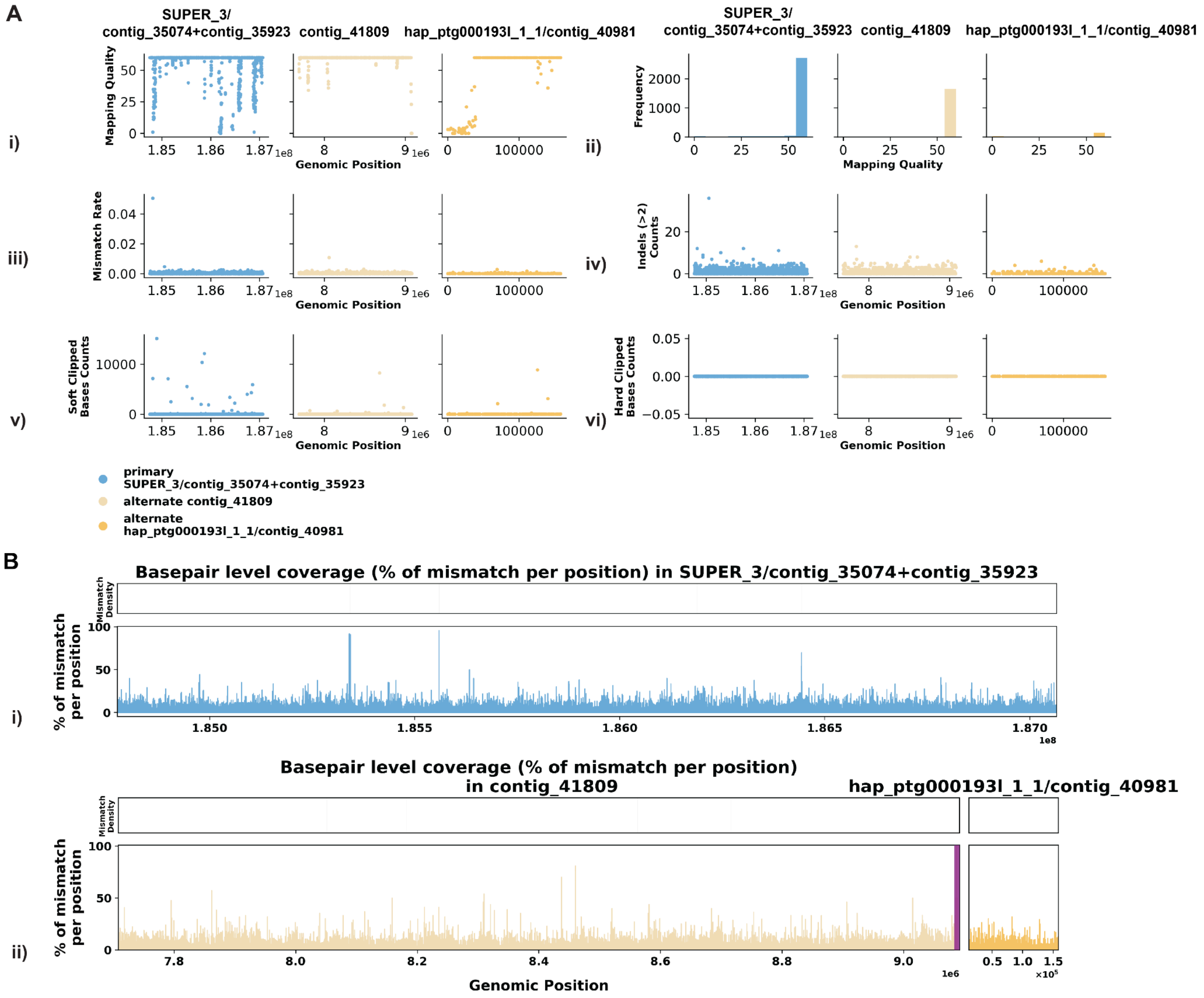

Figure S5. Additional Reassembly Analysis of IGH Locus Assembly Errors in *C. volans*. A. Summary statistics of the read alignment situation are depicted, showing mapping quality across IGH loci for both haplotypes, with blue representing the primary assembly and yellow the alternate. i) mapping quality across IGH loci for both haplotypes, with blue representing the primary assembly and yellow the alternate, ii) read mapping quality frequency, iii) mismatch rates of reads, and iv) number of reads with indels of consecutive length of at least 2 bp, v) count of soft clipped bases in each read, vi) count of hard clipped bases in each read, for both haplotypes across IGH loci. B. Reassembly IGH *basepair-oriented* mismatch rate across i) primary haplotype, ii) alternate haplotype; a heatmap above indicating the frequency of high mismatch rate base pairs, with darker colors denoting more frequent occurrences. Light red highlights positions covered by ≥5 reads with an error rate >1%, and purple bars indicate coverage breaks (coverage ≤2). C.

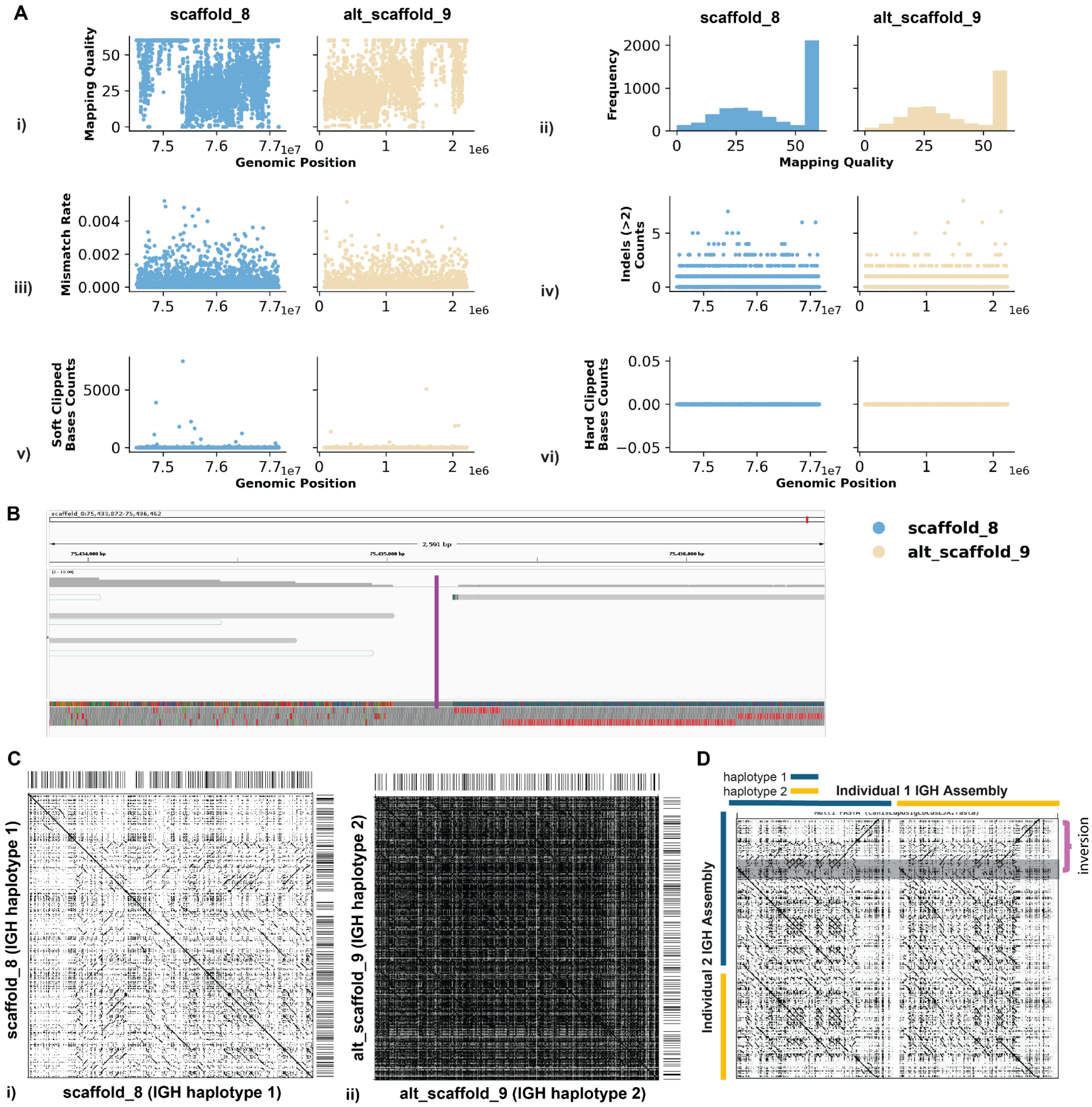

Figure S6. Additional Detailed Analysis of IGH Locus Assembly Errors in Mexican Gray Wolf (*C. l. baileyi*). A. Summary statistics of the read alignment situation are depicted, showing mapping quality across IGH loci for both haplotypes, with blue representing the primary assembly and yellow the alternate. i) mapping quality across IGH loci for both haplotypes, with blue representing the primary assembly and yellow the alternate, ii) read mapping quality frequency, iii) mismatch rates of reads, and iv) number of reads with indels of consecutive length of at least 2 bp, v) count of soft clipped bases in each read, vi) count of hard clipped bases in each read, for both haplotypes across IGH loci. B. IGV screenshot of the break in coverage C. Dotplots comparing gene locations and alignments are shown for i) primary vs primary and ii) alternate vs alternate haplotypes. D. Dotplots comparing *C. l. orion* assembly vs *C. l. Baileyi* assembly. Purple dashed line indicates the inversion observed.

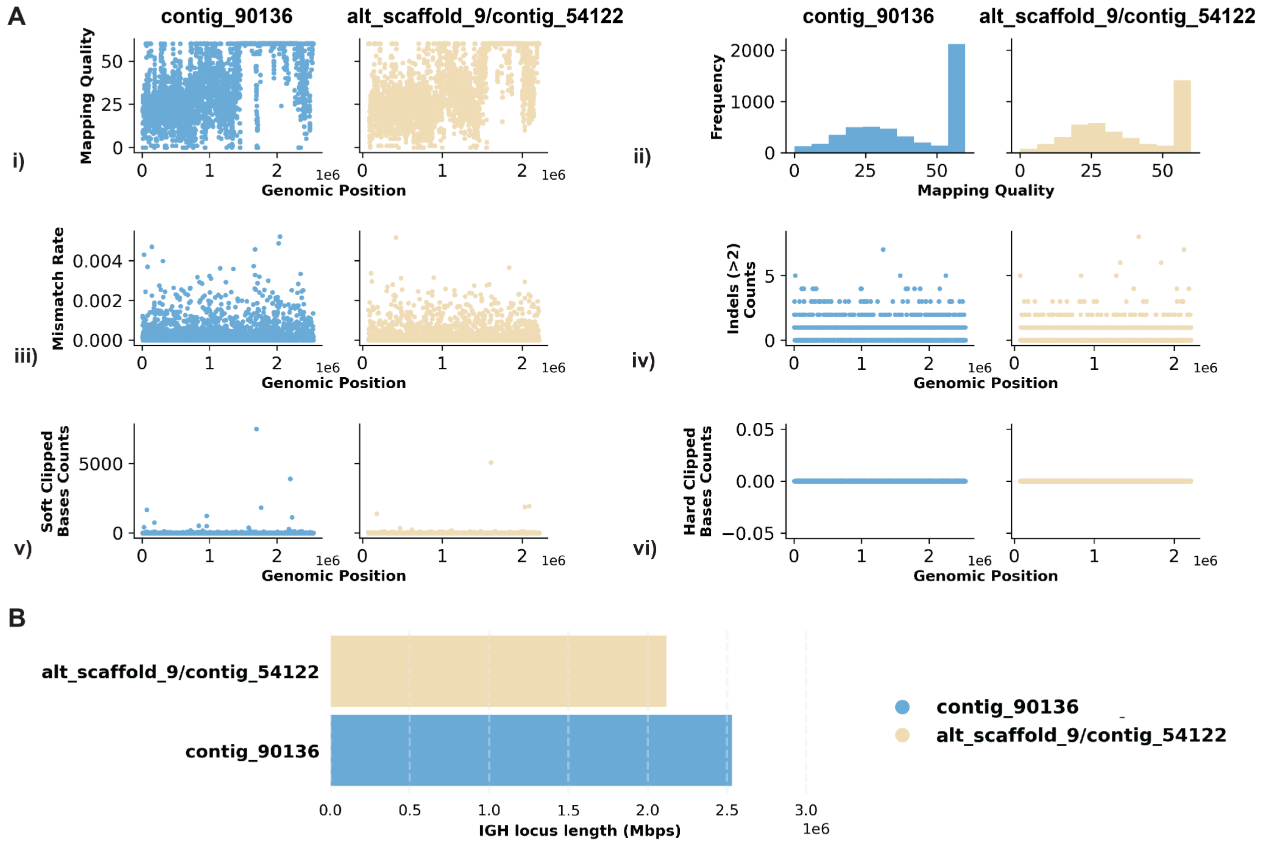

Figure S7. Additional Reassembly Analysis of IGH Locus Assembly Errors in *C. l. baileyi*. A. Summary statistics of the read alignment situation are depicted, showing mapping quality across IGH loci for both haplotypes, with blue representing the primary assembly and yellow the alternate. i) mapping quality across IGH loci for both haplotypes, with blue representing the primary assembly and yellow the alternate, ii) read mapping quality frequency, iii) mismatch rates of reads, and iv) number of reads with indels of consecutive length of at least 2 bp, v) count of soft clipped bases in each read, vi) count of hard clipped bases in each read, for both haplotypes across IGH loci. B. IGH locus length in both the primary and alternate assemblies are compared using bar charts.

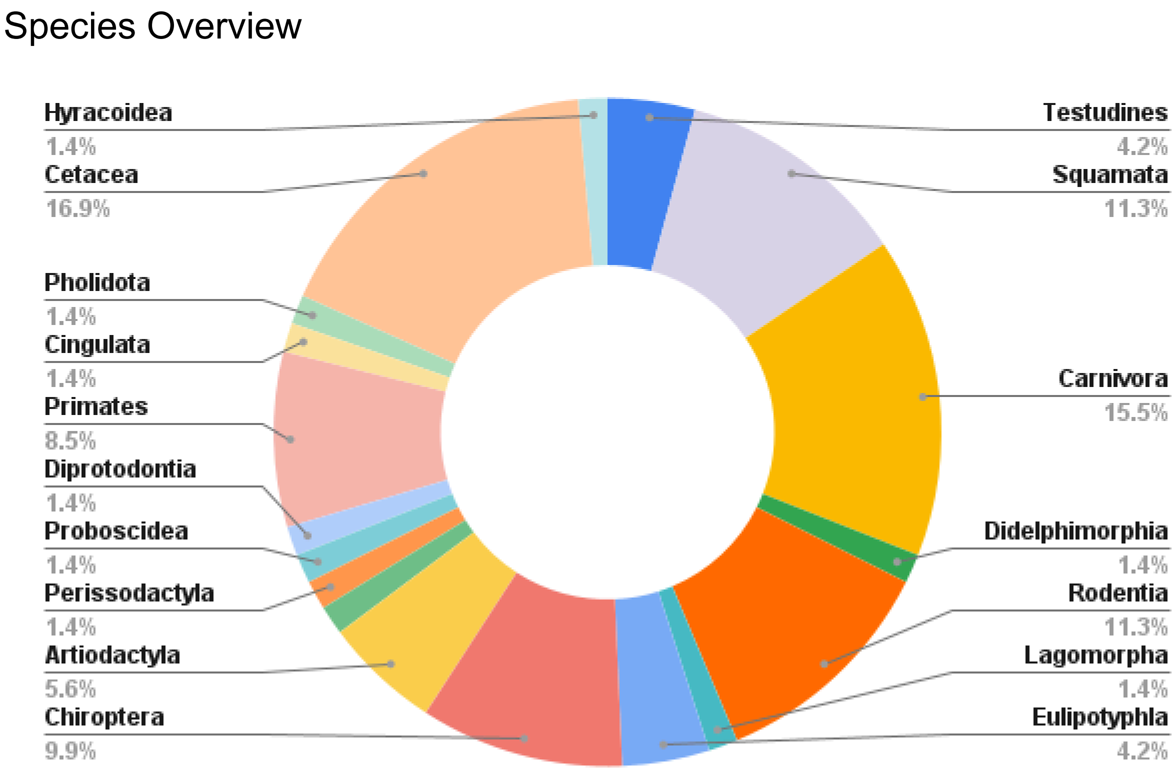

Figure S8. Pie chart summarizing the distribution of species by order.

Note S1: Comparison to CRAQ and Inspector

We ran CRAQ and Inspector on *C. l. orion* and *C. l. Baileyi* to evaluate their effectiveness. In the structural error output BED file provided by Inspector, the IGH loci for both individuals were missing, indicating that Inspector failed to detect structural errors in these regions. Similarly, CRAQ's results showed that the IGH loci for both individuals did not appear in the CSE (Clip-based Structural Errors) and CSH (Clip-based Structural Heterozygosity) outputs. Although CRAQ did identify these errors in its regional error output file, it classified them incorrectly as small-scale errors. This misclassification creates confusion and demonstrates the limitations of both tools in accurately detecting and categorizing errors in the IG loci. This underscores the necessity of developing CloseRead, as relying solely on existing tools like CRAQ and Inspector would have left us unaware of these critical inaccuracies. CloseRead provides a targeted approach to ensure precise detection and classification of errors, addressing the shortcomings of current tools.
